# Transportation and language access as crucial pillars for an immigrant-inclusive 21st-century food security program

**DOI:** 10.1017/S1368980023000575

**Published:** 2023-09

**Authors:** Jin K Park, Stella S Yi

**Affiliations:** 1 Harvard Medical School, Boston, MA, USA; 2 Department of Population Health, NYU Grossman School of Medicine, New York, NY 10016, USA

**Keywords:** Language access, Food insecurity, Transportation, Immigrant health

## Abstract

Though food insecurity has long been recognised to impact health, population-specific determinants of food insecurity have recently been studied systematically as an important public health concern. Indeed, while immigrant populations face particular challenges to equitable access to the food system, many of these concerns have not been systematically described. To this end, we critically review recent work that demonstrates the importance of transportation and language access as independent determinants of access to food for immigrant populations. Furthermore, we highlight proposals to mitigate barriers to access, including both academic and community-driven approaches to create overlapping institutional commitments for inclusive policymaking that meets the specific needs of diverse populations.

According to the US Department of Agriculture, in 2021, 13·5 million households in the US were food insecure, defined as lack of ‘consistent, dependable access to enough food for active, healthy living.’ During the ongoing Covid-19 pandemic, food insecurity has been of particular concern, given pandemic-driven closures of school nutrition programmes, unemployment and poverty. Internationally, there has been widespread recognition of nutritional inequality among minority communities. In the USA, there has also been much discussion on the need to design a new food security programme that has been battle-tested and takes seriously the lessons of the pandemic. The White House recently convened a conference on Hunger, Nutrition, and Health, with one of the five pillars of the national strategy being to ‘foster environments that enable all people to easily make informed, healthy choices’ and ‘invest in public education campaigns that are culturally appropriate and resonate with specific communities’^(1)^.

Furthermore, the Feeding America network recently published a Nutrition in Food Banking Toolkit, which outlined opportunities to transform the charitable food system to better address the needs of people facing hunger. A major theme of the report was intercultural competency of the charitable food system, acknowledging the importance of cultural food needs and choices of communities in ensuring respect for persons and minimising food waste^(2)^. Cultural competency of the charitable food system is particularly important given the diverse demographics of food-insecure populations and food pantry clientele^(2)^.

To design a 21st-century food security programme, policymakers should pay attention to specific burdens that exist for various communities, including for low-income, immigrant/ethnic minority populations, lending a new lens to how ‘cultural competency’ might be defined in this space. In this way, we aim to integrate recent work that has integrated the social and cultural determinants of health in efforts to strengthen the nation’s food safety system. Different populations face diverse structural barriers to food security. In this paper, we review two important yet overlooked determinants of food security for immigrant populations in particular—transportation and language—to uncover unmet needs and highlight tailored policy interventions as well as community-driven research.

## Food Security in the 21st Century

There has been significant research interest in identifying the determinants of food insecurity. One important way this work is done is by delineating the food environment of various populations through census tracts or geographical access points. Some have argued, in this context, that one of the most important determinants of food insecurity is geographic barriers to food such as lack of fresh food availability in a geographical area, often called ‘food deserts.’ There is accumulating evidence, however, demonstrating the inadequacy of this model for contextualising food insecurity for a variety of populations. For one, features that are often dispositive in explaining food insecurity in many contexts such as poverty and financial insecurity can be peripheral in many food desert models, especially if they are purely geographically conceived^(3)^. Indeed, even if food-insecure families had equal access to grocers, low-income families must make decisions between food, medicine, heating and housing that suppress food demand as a percentage of income^(3)^. The relationship between income support and food insecurity was demonstrated during the pandemic, when expansions of the Child Tax Credit for certain eligible families were associated with a significant reduction in food insecurity^(4)^.

Second, the ‘food desert’ model of food insecurity often relies on methods of measuring food environment that utilise a distance-based approach of proximity being a proxy for one’s food environment^(5)^. Recent literature on food shopping behaviour has demonstrated that most people do not shop in the same neighbourhood as their place of residence for their food. Clifton et al. have demonstrated through semi-structured interviews with low-income households that consumers’ understanding of their food environment was often more complex than geographical proximity^(6)^.

Lastly, the differentiated impact of food insecurity on various populations has been clear. Food insecurity is more prevalent in households with less than high school education, with at least one member with disability status, and amongst military veterans^(7)^. In 2021, rates of food insecurity were more prevalent among Hispanic and Black, non-Hispanic individuals^(7)^. Many national surveys assessing food insecurity generally are only conducted in English and Spanish, but not in other languages. A byproduct of this is an underrepresentation of limited English proficient communities – which is particularly salient for Asian American communities, where 34 % face limited English proficiency^(8)^. We recently demonstrated this to be the case, where national estimates from Census Pulse data collected in English and Spanish only reflected no disparities in food security for Asian Am^(9)^, our data collected locally (*n* 1270) in English and eleven Asian languages revealed food to be the number one concern during the COVID-19 pandemic for Asian American New Yorkers^(10)^. Because many immigrant populations have been cut-off by law from SNAP and other safety net programmes, understanding the barriers faced by immigrant populations will be important to design a durable and modern food security programme.

## Transportation and language access as social determinants of health and food access

Transportation has emerged in the health services literature as an important determinant for healthcare access in a variety of contexts. For instance, in a cross-sectional study that sought to determine significant predictors of chronic illness (age, race, language, gender, insurance, transportation, and food insecurity), transportation was identified as an important social need that was associated with adverse outcomes among patients with a chronic illness diagnosis^(11)^. Indeed, when transportation programmes have been implemented in conditions where transportation is a significant barrier to care, they have often determined that transportation is associated with improved access to care. A recent prospective study in a cohort of patients enrolled in an randomised controlled trial of hypertension found that housing and transportation displayed higher resistance to change than other social risk factors (food, clothing, healthcare, utilities, debt) and should be prioritised among social determinants for intervention^(12)^. For example, for immigrant/ethnic minority populations who travel further to receive culturally competent and in-language care, transportation access is an important determinants of health^(13–15)^.

The relationship between transportation and food access is more complex. In a 2009 consensus report to Congress, the USDA has determined that only a small proportion of food-insecure consumers are restricted in their ability to access affordable nutrition because they lack transportation, though this effect is greatly exacerbated in rural environments, where lack of transportation is ‘the most defining characteristic’^(16)^. As discussed in the previous section, consumers’ relationship with their food retail environment is greatly complicated by a variety of factors (proximity, work environment, delivery services), meaning that in large metropolitan areas, then, transportation is generally not thought to be an important determinant of food access.

An important question is whether the observed effects in large urban environments are associated with particular ethnic, racial or demographic groups. Yi et al., previously enumerated transportation to be a key conduit for urban immigrant communities to access culturally appropriate foods even in New York City. Yi et al. demonstrate that some immigrant populations—in particular Chinese Americans—display different food procurement patterns than the mainstream population, again similar with healthcare access, prioritising culturally specific grocery and produce items^(17)^. During the ongoing Covid-19 pandemic, researchers have documented the significant increase in food insecurity in the USA. One study found that between March and April 2020, 41 % of previously food-secure households became at risk for food insecurity, and that this increased risk was higher among racial minority populations as well as participants who were living with children and those who reported household incomes of below $100 000^(18)^.

Morales et al. demonstrated through survey data that Asian and Hispanic households were more likely to face transportation issues when purchasing food, and Black households were more likely to report that they could not afford to buy more food^(14)^. Indeed, Morales et al. speculate that Asian households who say that they are afraid to leave the home to procure food may face specific barriers such as lower income/education who do not have the knowledge to purchase groceries online, or from fear of leaving home. Furthermore, Asian food-insecure households had higher odds of reporting they did not leave to buy food because of transportation, mobility, or health issues – themes which were reflected in data collected in New York City^(10)^. Morales et al. also note that Hispanic food-insecure households also reported they were afraid or did not want to leave their homes to purchase food.

**Fig. 1:**
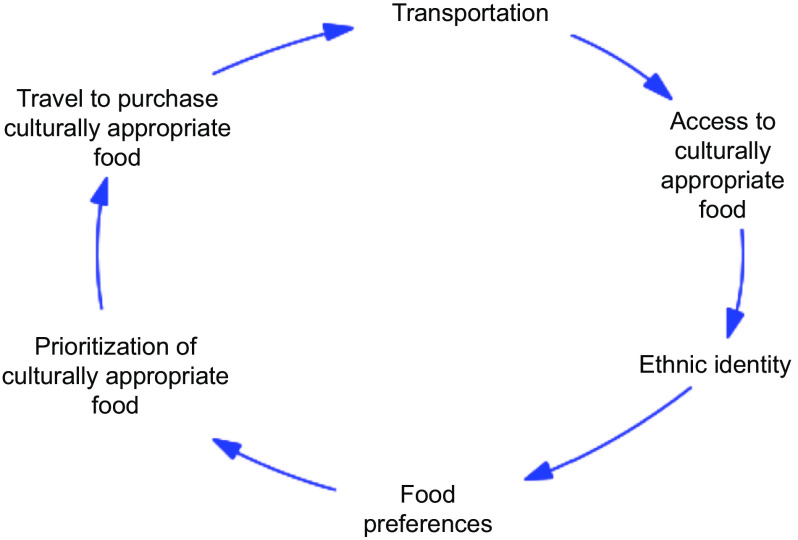
Transportation as a conduit to preserving ethnic identity and well-being

We posit that disruptions to transportation as observed during the COVID-19 pandemic not only impact access to food but may also have a synergistic effect of impacting well-being for immigrant communities by imparting a feeling of disconnectedness from one’s own communal identity (Fig. 1). Amid increased reports of anti-immigrant and anti-Asian violence, along with subsequent fear and shame felt in immigrant communities, there is a need to bolster programmes that improve social support and public trust. Addressing transportation access for immigrant communities could play a role in this process.

An important limitation of the studies discussed thus far is that they generally do not include data for particular racial-ethnic groups (e.g. Mexican, Chinese, etc.) nor do they analyse the effect size for non-citizen populations. Asian Americans have been shown to experience transportation barriers and health disparities, but those factors have not been linked to food insecurity, partly due to the lack of availability of disaggregated data and information on citizenship status.

In-language services are also important. A recent integrative review of thirty-three studies found that among the primary barriers to healthcare access for foreign-born individuals, English language proficiency (along with insurance status and citizenship/immigration status) are the drivers of healthcare access for foreign-born populations^(19)^. Disentangling language-based discrimination from other drivers of healthcare access such as citizenship status is especially important, since these determinants are not co-extensive. Yoo et al. show that the association between language discrimination and chronic disease status was stronger for Asian immigrants living in the USA > 10 years compared to recently arrived immigrants^(15)^. Parker et al. demonstrated that after adjustment of other variables, patient–physician language concordance was associated with improved glycemic control for limited-English language proficiency Latinos with type 2 diabetes^(20)^.

Language access acts as a key conduit for food in various contexts. Carney and Krause show through qualitative case studies that language access and immigration status are important determinants of food access for Mexican and Central American migrants in Santa Barbara county^(21)^. An in-depth qualitative study of adults living in East Harlem demonstrated that many Spanish- and Chinese-speaking adults’ shopping patterns were dictated by shopping at stores where food was cheapest or where the grocer spoke their language^(22)^.

## Recommendations: policy and community-based interventions

There are many important proposals in the literature to begin to address these challenges, and we describe these efforts below (Table 1).

1. *Integrate stable translation services for existing city and municipal food security programs.* Many cities and localities have food access programmes that serve significant numbers of non-English speakers. Certain components of the food security system, including school nutrition programmes—which serve as one of the most important bulwarks against child food insecurity—operate with foreign language services. Given that immigrant communities often live unintegrated from well-known food procurement networks, in-language resources on ethnic grocery stores and resources to navigate city and municipal food security programmes can be implemented. Increasing access to existing resources by providing translated materials, language support to enrol in benefits, and in-language recipes are all important developments. Another important responsibility of city and municipal food programmes is to pay attention to fear in immigrant communities due to concerns about public charge and leaving the home. In some cases, a trauma-informed lens will be important to develop tailored solutions for this population.
2. *Study transportation as a determinant of health, particularly as an important determinant of food and health for immigrant populations.* As described above, access to ethnic food and healthcare options for immigrant populations with low acculturation is an important source of health, well-being and sense of belonging in the USA^(14)^. Transportation in this way serves both as a linkage to food and also to cultural and ethnic sources of community integration and acculturation. It is therefore imperative to understand patterns of access and the role that transportation (e.g. personal, public, local transit systems) plays for immigrant communities in different geographic locations. Only with this formative knowledge can we then start to formulate solutions for connecting individuals to needed resources whether virtually (i.e. telehealth, online shopping) or through multi-sector partnerships to address community transportation options.
3. *Encourage immigrant participation in experimental stipend programs to curb food insecurity.* The past few years have seen significant nation-wide experimentation in unconditional stipend programmes to support individuals in and around care. Recent evidence has shown that unconditional stipend programmes led to health-promoting purchases^(23)^. Since many immigrants are excluded from SNAP, community-engaged approaches will be important to work with these communities.
4. *Encourage broader public participation in research efforts.* As we have reviewed, there is significant shortcomings with many models of food insecurity, since they rely on a geographical understanding of individuals’ food environment. Furthermore, as Vu et al. argue, the lack of research into the determinants of food insecurity in immigrant and refugee populations have made it difficult to create tailored policy solutions for this population^(24)^. As research efforts are underway to understand and model food security and access in ways that reflect lived realities, investigators and funding bodies can encourage complex systems thinking in food security research. For instance, participatory mapping strategies that include a diverse array of community stakeholders can ensure that diverse needs are met^(25)^. Funding sources can encourage complex systems thinking. Community-based outreach efforts will be important to building trust in research efforts and institutions. Grants such as the USDA Community Food Projects Competitive Grant can foster innovative efforts to involve broader public participation to create tailored interventions for diverse communities.

**Table 1:** Stakeholders and recommendations for an immigrant-inclusive food security programme

Recommendation	Potential examples	Key stakeholders
Integrate stable translation services for existing city and municipal food security programmes	Create and increase access to translated materials for existing benefits	Government agencies, community-based organizations
	Forge partnerships with community-based organizations to integrate immigrants to governmental and NGO-driven food procurement networks	Government agencies, community-based organizations, researchers
	Bolster social support for immigrant communities due to fear of leaving the home as well as hesitations about public charge	Government agencies, community-based organizations
Study transportation as a determinant of health, particularly as an important determinant of food and health for immigrant population	Investigate how transportation acts as a key determinant of health for immigrant populations, with special attention to ethnically and linguistically familiar food and healthcare options as an avenue for community integration	Researchers, government agencies
	Whenever possible, report disaggregated results from studies to ensure interventions can be tailored to population-specific needs	Researchers, government agencies
Encourage immigrant participation in experimental stipend programmes to curb food insecurity	Integrate community-based organizations in experimental stipend programmes to encourage immigrant participation	Researchers, funding agencies, local governments
Encourage broader public participation in research efforts	Include as many participants as possible from diverse backgrounds in participatory mapping strategies for understanding food environment	Researchers
	Fund research efforts that integrate various components of the health system to understand the complexity of the health system	Funding bodies

To close, the renewed attention to the importance of nutrition and access to healthy food is heartening. In nations around the world where nutritional inequality is a major public health challenge, these recommendations can help to build trust among various impacted populations and bolster system-wide reform. In this paper, we have focussed on the specific impacts of food insecurity on migrants and refugees– who make up 14 % of the US population, and are a significant portion of the workforce of other nations. We have focussed here on transportation and language access, but further study of other determinants, particularly as they impact populations on an intersectional basis, are needed.
